# Why We Should Look at Dinner Plates: Diet Changes in Cancer Patients

**DOI:** 10.3390/curroncol30030205

**Published:** 2023-02-23

**Authors:** Katja Döring, Lara Wiechers, Jens Büntzel, Judith Büntzel

**Affiliations:** 1 Institute for Diagnostic and Interventional Neuroradiology, Hannover Medical School, 30625 Hannover, Germany; 2 Department of Radiation Therapy and Radiation Oncology, University Hospital Göttingen, 37075 Göttingen, Germany; 3 Department of Otolaryngology, Head Neck Surgery, Südharz Hospital, 99734 Nordhausen, Germany; 4 Department of Hematology and Medical Oncology, University Hospital, Robert-Koch-Straße 40, 37075 Göttingen, Germany

**Keywords:** malnutrition, body mass index, prognosis, screening tool, dietary changes

## Abstract

Objective: Malnutrition is often underestimated in the context of cancer therapy: the dietary trends initiated by patients after diagnosis are usually neither known to nor evaluated by the medical staff. Here, we propose a combined screening instrument evaluating malnutrition and dietary trends. Methods: The validated screening tool NRS-2002 was combined with a four-item questionnaire assessing whether (1) patients preferred certain foods, (2) avoided certain foods, (3) used dietary supplements or followed a special diet since the time of cancer diagnosis. The screening tool was routinely used by cancer patients in the daily practice of three oncological departments. The presented analysis was performed retrospectively and anonymized. Results: Overall, 102 cancer patients undergoing systemic therapy (CP), 97 undergoing radiation therapy (RP), and 36 head–neck cancer patients (HNP) were screened. The CP cohort showed a higher rate of malnutrition (50.00%) than the HNP (28.13%) or RP (26.80%) cohort. Overall, diet changes were observed in 33.63% of all patients. Avoiding meat, stimulants, or hard and edgy food was often mentioned in free text answers, while patients reported a preference for fruit and vegetables. Nutritional supplements were used by 28.76% of the patients. While dietary changes were common, only 6.64% of the patients mentioned adhering to a specific cancer diet. Conclusion: Malnutrition is still underestimated nowadays. Diet trends, especially avoiding certain foods, are common in cancer patients, while adhering to a specific cancer diet is an exception. Diet trends should be assessed and addressed to avoid or aggravate malnutrition.

## 1. Introduction

Malnutrition is a common problem in cancer patients, often based on unintentional weight loss due to inadequate nutrient intake or uptake [1]. Nutritional status of patients differs between cancer entities and is also influenced by side-effects of oncological therapy [2]. Approximately 15–40% suffer from malnutrition at the disease’s onset, and the prevalence further increases up to 40–80% in patients undergoing oncological therapy [3].

Malnutrition affects several aspects of cancer treatment and outcome. Malnourished patients show an increase in treatment toxicity and a worse overall survival compared to well-nourished patients undergoing the same treatment [2]. Nutritional problems leading (in the worst case) up to cancer cachexia should be viewed as a continuum, starting from initial signs and symptoms of anorexia to cachexia or even refractory cachexia [4]. It is well known that the efficacy and impact of nutritional interventions are related to the timing of support, with the best results obtained with early intervention or prehabilitation [5,6].

Accordingly, it is important to regularly screen cancer patients for malnutrition during the various phases of treatment and the disease. Nutritional status is fluid and changes overtime. Not only cancer entity, as mentioned above, but also tumor stage, treatment type and setting, and concomitant diseases influence the patient’s nutritional needs. This argues for the necessity to continuously assess nutritional status [2]. Several standardized tools have been established for malnutrition screening. The European Society for Clinical Nutrition and Metabolism suggests to use the Malnutrition Screening Tool (MUST), the Nutritional Risk Screening (NRS-2002), or the Mini Nutritional Assessment [7]. The scored Patient-Generated Subjective Global Assessment (PG SGA) tool offers a time-intensive instrument, which was validated for tumor patients [8]. Nevertheless, it is not been established in the clinical practice. So, the current ESPEN practical guidelines commonly endorse to regularly evaluate nutritional intake, weight changes, and BMI at the time of cancer diagnosis and argue for subsequent reevaluations during cancer treatment [9].

While cancer and oncological treatment may lead to malnutrition [2], we should also consider the patients themselves. A small cross-sectional study in Germany revealed that up to 70% of the patients surveyed had or planned to change their diet. These dietary changes themselves may have also an effect on (mal)nutrition [10]. This argues to not only assess for malnutrition but also dietary changes. We recently proposed a short questionnaire to detect cancer diets [11]. We propose to combine the latter with the Appendix A NRS-2002 [12] and present here a cohort of 235 cancer patients assessed for both malnutrition and dietary changes.

## 2. Materials and Methods

Patients suffering from a solid or hemato-oncology cancer were enrolled in a non-interventional, anonymous, cross-sectional, retrospective study. Patients were routinely screened at the time of admission to inpatient care as a part of the clinical (admission) routine. The following departments participated: Institute of Radiation Oncology and Radiation Therapy of the University Medical Center Göttingen, Department for Hematology and Medical Oncology of the University Medical Center Göttingen, and the Department of Otorhinolarnygology of the Südharz Hospital in Nordhausen between September 2021 and June 2022. The retrospective analysis of data was approved by the local ethics committee of the University Medical Center (approval number: 22/6/22).

All included patients were screened with the NRS2002 and a four-item questionnaire proposed to identify dietary changes in cancer patients [11,12]. The NRS-2002 is a validated tool which offers a pre-screen, evaluating general low nutritional intake, weight loss, low BMI, or disease severity. In case of a positive pre-screen, the actual screening combines a more detailed assessment of weight loss/nutritional intake and disease severity and age [10]. The four-item questionnaire includes the following questions: (1) “Do you dispense or avoid specific food?”, (2) “Do you prefer specific food?”, (3) “Do you take additional supplements?”, and (4) “Do you follow a specific diet strategy?”. Patients are asked to answer if changes in nutritional behavior occurred after getting the diagnosis of cancer [11]. “Dietary changes” were defined as changes (avoidance/preference) in consuming specific foods, while a “specific cancer diet” was defined as the conscious decision to follow a specific dietary regimen.

If patients answered with “yes” concerning questions of avoiding or preferring certain foods, such as additional supplements or specific diet strategies, free text answers were written down. The free text answers of the patients concerning nutritional changes, cancer diets, or nutritional supplements were retrieved from questionnaires and translated into English. Answers were further summarized in categories (e.g., “red meat” and “beef” were summarized as “meat”). Each answer was considered equally. Instead of pie diagrams, we chose to draw word clouds to depict recurring main topics of patients. Word clouds were drawn using the free online software https://www.wortwolken.com/ (accessed on 9 January 2023). The larger the words are presented in each word cloud, the more often this specific answer was given by patients.

Additional data on patients’ gender, cancer entity, and age were evaluated. When available, albumin and C-reactive protein (CRP) data were evaluated to calculate the modified Glasgow prognosis score (mGPS) [13]. Data were analyzed using an Excel Spreadsheet (Excel 2013) and GraphPad Prism (GraphPad Software, Version 8.0). Cohorts of patients were both analyzed separately by cohort. All cohorts were pooled for entity-specific subgroup analysis to reach a sufficient sample size for statistical analysis. If patient numbers of a single entity were not sufficient for subgroup analysis, single entities were summarized as an entity group: hematological malignant (lymphoma, acute myeloid leukemia, multiple myeloma, myeloproliferative neoplasia, chronic lymphatic leukemia, myelodysplastic syndrome, and chronic monocytic leukemia), hematology benign (anemia, idiopathic thrombocytopenic purpura, and others), lung cancer (non-small cell lung cancer, small cell lung cancer, and others), head–neck cancer (larynx carcinoma, oropharynx carcinoma, hypopharynx carcinoma, nasopharynx carcinoma, and others), other gynecological cancers (cervical and vulva carcinoma), uroonclogy (prostate carcinoma, urothelial carcinoma, and renal carcinoma), and upper gastrointestinal tract (esophagus carcinoma and others).

Due to the sample size, Fisher’s Exact test instead of Chi-Square test was chosen for analyzing independence of dichotomous parameters. Pearson’s r was applied for correlation analysis. Patients with a positive pre-screen (NRS2002) were included for correlation analysis. We a priori planned to test for correlation between the NRS-2002 score (numerical values) and the albumin, C-reactive protein, or mGPS. A *p*-value < 0.05 was considered significant for the statistical tests applied.

## 3. Results

### 3.1. Malnutrition Is Common amongst Cancer Patients

Overall, we were able to pool data from three cohorts: the radiation cohort comprised 97 patients; the hematology/medical oncology cohort, 102 patients; and head–neck cohort, 36 patients. A total of 235 patients were included for data analysis. Of those, 144 identified as male and 91 as female. Median age was 65.64 years (range 29.43–88.35 years). We also stratified for cancer entities (Table 1).

Overall, more than 50% of the patients (131/235) included showed positive results in pre-screening and subsequently underwent the main screening of the NRS2002 tool. Here, 86 patients suffering from malnutrition were identified. Taken together, we observed malnutrition in 36.60% (86/235) of all patients. Subgroup analysis (Table 2) was possible for the following five groups: breast cancer, hematology (malignant), head–neck cancer, lung cancer, and urooncology. Malnutrition rates were: 11.76% (2/17, breast cancer), 48.44% (31/64, hematology-malign), 31.82% (14/44, head neck cancer), 43.60% (17/39, lung cancer), and 47.06% (8/17, urooncology). Compared to the whole cohort, patients suffering from malignant hematological neoplasia showed significantly higher malnutrition rates (Fisher’s exact test, *p* = 0.033, Table 2).

We observed a trend towards lower albumin levels in these patients (Pearson’s r = −0.218, *p* = 0.071), while higher CRP levels were associated with a higher NRS2002 score (Pearson’s r = 0.223, *p* = 0.064). No significant correlation was found between NRS2002 and mGPS (Pearson’s r = 0.108, *p* = 0.376).

### 3.2. Diet Changes Are Common amongst Cancer Patients, Not Specific Cancer Diets

Only a minority of our patients followed a specific (cancer) diet: 6.64% (15/226 patients). Two items of our cancer diet questionnaire assessed whether patients avoided or preferred certain foods after cancer was diagnosed. A total of 33.63% (76/226) of the patients changed their nutritional behavior by either avoiding (27.43%, 62/226 patients) or preferring (20.80%, 47/226 patients) certain foods. Stratification showed that overall diet changes were similar between entities. However, patients with malignant hematological neoplasia significantly more often preferred certain foods (Fisher’s exact test, *p* = 0.017) than other cancer patients, and head–neck cancer patients tended to avoid specific foods more often (Fisher’s exact test, *p* = 0.089).

Overall, most patients avoided meat products (38.46%, 30/78 answers), stimulants (16.77%, 13/78 answers), edgy or hard foods (11.54%, 11/78 answers), and sugar/carbohydrates (11.54%, 11/78 answers). Only a minority avoided milk products (6.41%, 5/78 answers), spicy/sour foods (5.13%, 4/78 answers), or others (10.26%, 8/78 answers).

Amongst preferred food were fruits (25.00%, 23/92 answers), vegetables (25.00%, 23/92 answers), and carbohydrates (16.30%, 15/92 answers). Less commonly preferred foods were milk products (6.52%, 6/92 answers) and fish/meat products (6.52%, 6/92 answers); A total of 20.65% of foods (19/92 answers) was subsumed as “other”. Overall, we observed a subgroup of patients with food preferences showing an adaption towards mucositis or dysphagia (17.39%, 16/92 answers, categorized as “dysphagia nutrition”).

Supplements were used by 28.76% (65/226) of the patients. Here, we observed significantly higher user rates amongst breast cancer patients (70.59%, 12/17 patients; Fisher’s exact test, *p* = 0.002). In contrast, head–neck cancer patients had the lowest user rates within our cohort (11.90%, 5/42 patients; Fisher’s exact test, *p* = 0.001). For data concerning different entities and cohorts, refer to Table 3 and Table 4.

Most commonly used supplements were vitamins (50.35%, 72/143 answers) and micronutrients (32.17%, 46/143 answers). Amino acids/proteins (3.50%, 5/143 answers) and fatty acids (2.80%, 4/143 answers) were less commonly used. As vitamins and micronutrients were the most commonly used supplements, we differentiated the answers. Overall, patients used the following vitamins: vitamin D (23.01%, 26/113 answers), vitamin B (including vitamin B12, vitamin B6, “vitamin B complex” preparations; 14.16%, 16/113 answers), vitamin C (8.85%, 10/113 answers), or others (14.16%, 16/113 answers). Concerning micronutrients, patients used magnesium (18.58%, 21/113 answers), selenium (7.08%, 8/113 answers), iron (6.19%, 7/113 answers), zinc (4.42%, 5/113 answers), or others (3.54%, 4/113 answers).

**Figure 1:**
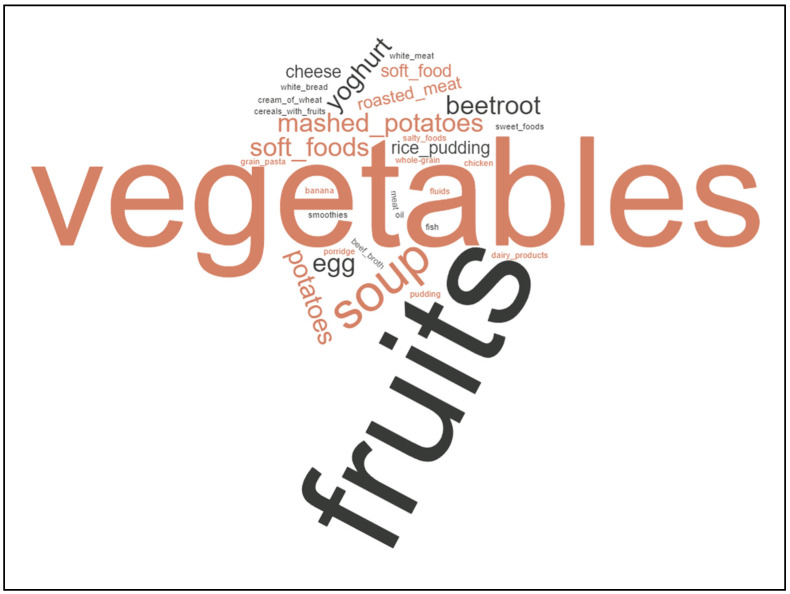
Word cloud depicting patients’ free text answers showing preferences towards fruit, vegetables, and soft foods such as mashed potatoes or soup.

The foods standing out as the most often preferred were “fruits”, “potatoes”, “mashed potatoes”, “soup”, or “no hard foods” (Figure 1).

**Figure 2:**
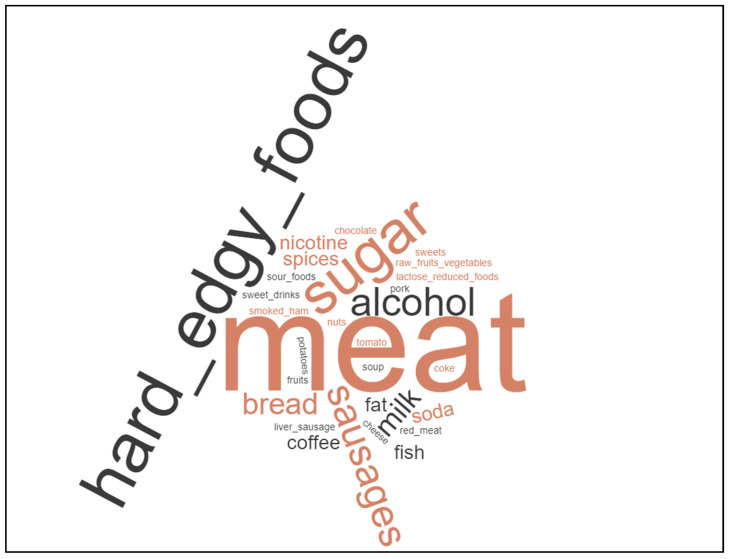
Word cloud depicting patients’ free text answers showing that patients especially avoided meat, hard and edgy foods, and sugar.

The latter three may hint towards dysphagia and adaptation towards side-effects in patients. Patients avoided mostly “meat” or “sausages and cold cuts”, “sugar”, “alcohol”, “bread”, and “hard foods” (Figure 2).

**Figure 3:**
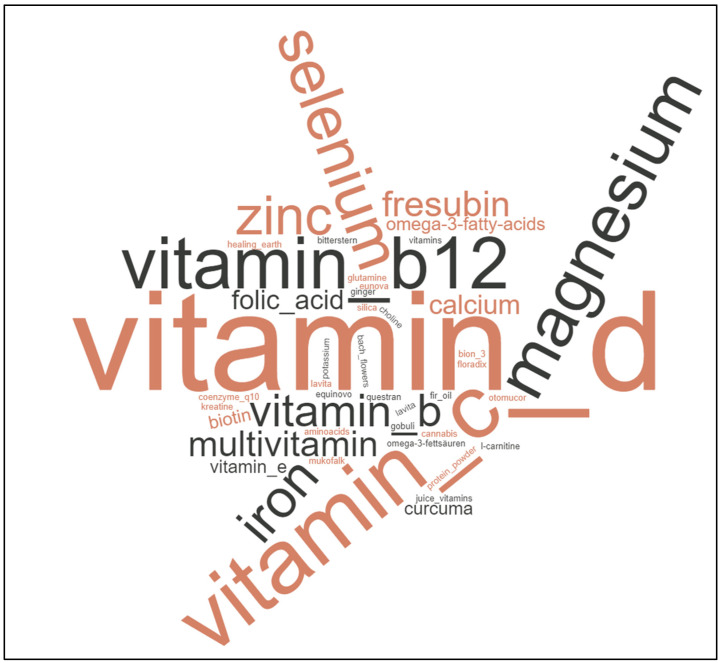
Word cloud depicting patients’ free text answers concerning use of nutritional supplements. Vitamins such as vitamin D or B12 and micronutrients such as magnesium were popular.

Here again, our patients’ answers may hint towards dysphagia (hard foods) and a reduced protein uptake (less meat). Answers on what diet changes happen after being diagnosed with cancer may offer insight on the causes of malnutrition. Amongst the supplements, vitamin D, vitamin B12, vitamin C, and magnesium are the most often used substances (Figure 3).

## 4. Discussion

Since 2006, the worldwide Nutrition Day is an established benchmark program analyzing the role of nutrition in clinical practice of the participating health care systems. In 2021, 11% of the participating German hospitals did not screen for malnutrition, another 8% used visual assessment, 30% used BMI or weighing, and only 32% used the application of a screening tool for malnutrition [14]. Units treating cancer patients mainly considered nutritional treatment “when the patients asked” or at a weight loss of >10%. Less than the half of all cancer care units (46%) routinely recognized the need for nutritional treatment [14]. These data stand in contrast to the current ESPEN recommendations to routinely screen cancer patients’ nutritional intake, weight changes, and BMI at disease onset and to subsequently reevaluate nutritional status during cancer treatment [9]. The ESPEN suggests several different validated screening tools to detect malnutrition or patients at risk of developing malnutrition such as the PG-SGA, NRS-2002, MUST, or, in the elderly, the Mini Nutritional Assessment (MNA) [4]. The MUST was developed with the aim of community use, keeping in mind that here serious confounders of the effects of undernutrition are relatively rare. In contrast, the NRS-2002 focuses on detecting undernutrition or the risk of developing undernutrition in an in-patient setting and therefore assesses not only the nutritional components, such as the MUST, but also the disease severity [15]. The PG-SGA has been previously validated for cancer patients [4]. The high diagnostic performance of the PG-SGA in cancer patients [16] comes unfortunately at the cost of the time required for screening. This makes the PG-SGA difficult to integrate into daily clinical routine. Considering the latter and that the NRS-2002 is predominantly used in German hospitals [14], we decided to apply this screening tool in our cross-sectional study.

The Global Leadership Initiative on Malnutrition (GLIM) established the “GLIM criteria” to ensure a well-defined, common definition of malnutrition in 2016. Amongst these are non-volitional weight loss, low BMI, reduced muscle mass, decreased food intake or assimilation, and inflammation or disease burden [17]. The NRS-2002 is valuable tool to assess for a majority of these criteria. However, neither NRS-2002 nor the GLIM criteria are able to give information on dietary changes of patients, which may also have an impact on weight-loss, food intake, or assimilation. Similar information will be received by using the PG SGA as a shortened screening instrument. Adding four items [11] to our nutritional screening, asking whether patients avoided or preferred certain foods from the time of cancer diagnosis, used micronutrients, or even followed a specific (cancer) diet, adds valuable information to the patients’ history.

We know that malnutrition affects up to 75% of cancer patients. The prevalence and resulting variation is determined by cancer-related (type, stage, and treatment), demographic (age), and social factors [2]. Overall, we used our modified screening tool to survey 235 patients. Half of all the patients showed a positive pre-screen and were referred to in-depth screening. In total we observed a rate of 36.60%, which is within the range of the published data [2,18]. Similar to previous studies [18,19,20,21], we observed different rates of malnutrition between cancer entities from 11.76% in breast cancer patients to 48.40% in patients with hematological neoplasia.

Difference in malnutrition rates between entities—ranging in our cohort from 11.76% in breast cancer patients to 48.44% in patients with hematological neoplasia—is not an uncommon phenomenon. Malnutrition rates of lung (43.60%) and head–neck cancer (31.82%) in our study were similar to the previous data [20,21,22,23,24]. For patients with hematological neoplasia, the malnutrition rate in our study was higher than the data found in the literature [18,19,24,25]. Our higher rate might be explained by the facts that the three studies screening inpatients [18,19,24] did not use a screening tool but information on BMI and weight loss to assess for malnutrition and that [25] applied the PG-SGA on a cohort of (fitter) out-patients.

Overall, our data shows the high risk and prevalence of malnutrition in specific groups of patients. This is not surprising as cancer therapy differs between entities and show different patterns of side effects, be it locoregional impairments (e.g., due to radiation or surgery) or side effects of systemic therapy (e.g., anorexia, oral discomfort) [26,27]; these may influence the development of malnutrition. Further, we should consider that malnutrition could also be influenced by tumor-induced activation of inflammatory pathways, which then may cause anorexia, altered metabolism, and involuntary loss of lean and fat mass, eventually leading to cachexia [28,29,30]. Very recently mass spectrometric analysis of blood sera even showed an entity of specific metabolomics profile in patients with upper gastrointestinal cancer [31]. Similar to these results, our study supports the previously published concept that each cancer entity is characterized by an entity-specific risk of accompanying malnutrition [2]. Oncologists should consider this entity-specific risk as malnutrition and cachexia are strong prognostic markers for unfavorable clinical outcomes [32]. Overall, this demonstrates the necessity to pay special attention to vulnerable subgroups as early as possible. Our data argue for paying increased attention towards the prognostic impact of malnutrition and low muscle mass on those undergoing treatment of hematological neoplasia, lung, or head–neck cancer and on patients suffering from urooncological neoplasia.

While activation of inflammation pathways especially characterizes cachexia [28,29,30], we did not observe a significant association of C-reactive protein with malnutrition. Similarly, albumin status did not correlate with the results of the NRS-2002. This result fits with the analysis of a recent review that argues to consider both laboratory parameters as potential prognostic markers for overall survival but not for malnutrition [33].

Laboratory parameters as well anthropometric data do not offer insights in dietetic behavior of cancer patients. However, the latter may have a direct influence on (developing) malnutrition. The usefulness or also the harm caused by adhering to a specific cancer diet is a controversy discussed in the literature [34,35,36]. However, the community is aware that patients show interest in specific cancer diets that diverges from the official dietary guidelines of, for example, the American Cancer Society or the American Institute for Cancer Research/World Cancer Research Fund [35]. Despite the knowledge about patients’ interest in cancer diets, there is no tool to screen for adherence to a specific diet. Neither the NRS-2002 nor the MUST catch shifts in our patients’ dietary behavior. Both tools only register malnutrition, which might be a subsequent consequence of adhering to a specific diet. Therefore, our group proposed a short four-item questionnaire to assess for both specific cancer diets and dietary changes/intake of nutritional supplements [11]. Data on users of specific cancer diets are, to our knowledge, not available. Zick et al. proposed that the rate of patients using complementary and alternative medicine (CAM) might correspond with the rate of patients adhering to specific dietary regimen [35]. As 40–90% of cancer patients use CAM [37,38,39,40], one would expect high rates of patients following a specific diet. However, only 6.64% of our cohort adhered to a specific diet. This low number might be explained by (1) the popularity of cancer diets is largely overestimated and (2) the rate might have been higher if our cohort had included more breast cancer patients, who are known to be more interested in CAM [39,41,42]. Summarizing, the low rate of patients using specific cancer diets argues for analyzing diet changes. Those are subtler, but may also have an impact on nutritional status (e.g., on protein intake if patients avoid meat without replacing proteins through alternative sources). We know that cancer patients often change or plan to adapt their dietary behavior [10,43]. Studies also showed that dietary changes of patients are not necessarily in line with the official dietary recommendations [35,43], and this may be, therefore, potentially an underlying cause of developing malnutrition. Dietary changes involve both avoiding and preferring certain foods. While another smaller German study described that up to 70% of surveyed out-patients changed or planned to change their diet [10], we observed that only a third of the patients in our cohort reported dietary changes. By comparing the data between [10] and our study, we may however appreciate common, recurring topics amongst patients: e.g., a higher intake of fruits and vegetables, eating less meat, or avoiding sugar or carbohydrates. Our survey also offers a new, previously underrated insight—a small group of patients declared avoiding hard or edgy food. This insofar is interesting as this uncovers that dietary changes are not always due to the motivations of benefitting health or actively contributing to therapy [10] but are also an adaptation to current needs or impairments (e.g., not eating hard foods when suffering from oral discomfort). Our data shows that the presence of specific cancer diets is overestimated and the pitfalls of dietary changes are underestimated. Screening for malnutrition using established tools like the NRS-2002 should be complemented by taking patients’ nutritional history assessing patterns of preference and avoidance. Our short four-item questionnaire offers a fast option for screening and recognizing patients that may require further counseling or nutritional intervention. Additionally, our fourth question on usage of nutritional supplements offers additional information to the medical staff and enables them to counsel patients, whether supplement intake can have negative effects on cancer therapy (e.g., vitamin E and radiation therapy in head–neck cancer [44]).

Overall, our results confirm that patients develop an awareness of their own diet and adapt their dietary behavior. A needs-based, individualized assessment of the nutritional status is necessary for individual counseling and intervention. Unfortunately, the actual attention given to nutritional status is far from the standard required in oncology management [45].

### Limitations

We present a retrospective cohort study with 235 patients only, undergoing either systemic (chemo-) therapy or radiation therapy. All cancer entities were included. Subgroup analysis was only possible for five entities, and even here, especially the numbers for patients with breast cancer and urooncological neoplasia were relatively small, which may have led to over- or underestimating rates of malnutrition and also dietary trends in these subgroups. Data analysis was only descriptive; therefore, we did not consider adjusting for multiple testing.

Our screening tool is reductive and gives only an input for detailed counselling. Impact factors as surgery, irradiated fields, or GI diseases were not asked. The screening tool does not substitute any individual assessment and counselling.

Furthermore, the questionnaire was used for patients under therapy. So, we have no sufficient information for longtime trends in cancer survivors. Nutritional trends and specific diets would be interesting terms in follow-up investigations too. Subgroup analysis of entities gives us first impressions on which patient groups are more prone to changing their diet or using supplements. Data of subgroup analysis however should be interpreted carefully due to a, sometimes, small case number.

## 5. Conclusions

The spectrum and extent of malnutrition in oncological patients varies depending on the cancer entity. The NRS-2002 is a good tool to recognize malnourished patients; however, dietary trends and changes are not considered by established screening tools.

Our four-item questionnaire is able to detect such nutritional trends, showing that the importance and prevalence of patients adhering to specific cancer diets is overestimated and the relevance of dietary trends and their potential influence on malnutrition are underrated. Therefore, we argue for adding a short screening of dietary trends to the standardized screening for malnutrition, which then offers a common ground for personalized counseling and intervention.

## Figures and Tables

**Table 1:** Clinical characteristics.

Total		235
**Gender**	Male	144
	Female	91
**Age**	Median (range) [years]	65.64 [29.43–88.35]
**Cohort**	Radiation	97
	Hema/Onco	102
	Head–neck	36
**Entity**	Breast	17
	Other gynecological	6
	Urooncology	17
	Head–neck	46
	Lung	39
	Colorectal	8
	Upper gastrointestinal tract	9
	Cancer of unknown primary	4
	Hematology malignant	64
	Hematology benign	10
	Other	15

**Table 2:** Malnutrition in cancer patients assessed using the NRS2002.

Entity	Pre-Screen Negative [N]	Pre-Screen Positive [N]	*p*-Value
Breast	13	4	**0.0093**
All	91	127	
Hematology malignant	27	37	0.7685
All	77	94	
Head–neck	24	20	*0.1338*
All	80	111	
Lung	16	23	0.7257
All	88	108	
Urooncology	5	12	0.3106
All	99	119	
**Entity**	**NRS2002 < 3** **[N]**	**NRS2002 ≥ 3** **[N]**	***p*-Value**
Breast	2	2	0.5829
All	35	84	
Hematology malignant	6	31	**0.0326**
All	31	55	
Head–neck	6	14	1.0000
All	31	72	
Lung	6	17	0.8023
All	31	69	
Urooncology	4	8	0.7516
All	33	78	

Significant *p*-values were depicted in bold.

**Table 3:** Diet changes: subgroup analysis of entities. Each sub-group was compared against all other cases.

Entity	Diet Change: Yes	Diet Change: No	*p*-Value
Breast	4	13	0.4352
Others	71	137	
Hematology malignant	25	38	0.2721
Others	51	112	
Head–neck	18	26	0.2874
Others	58	124	
Lung	10	29	0.2698
Others	66	121	
Urooncology	4	13	0.4352
Others	71	137	
**Do You Prefer Specific Food?**			
**Entity**	**Prefer Foods: Yes**	**Prefer Foods: No**	***p*-Value**
Breast	2	15	0.5354
Others	45	164	
Hematology malignant	20	43	**0.0169**
Others	27	136	
Head–neck	11	33	0.5345
Others	36	146	
Lung	7	32	0.8284
Others	40	147	
Urooncology	1	16	0.1328
Others	46	161	
**Do You Dispense or Avoid Specific Food?**			
**Entity**	**Avoid Foods: Yes**	**Avoid Foods: No**	***p*-Value**
Breast	4	13	1.000
Others	58	151	
Hematology malignant	17	46	1.000
Others	45	118	
Head–neck	17	27	0.0889
Others	45	137	
Lung	9	30	0.8307
Others	43	164	
Urooncology	4	13	1.000
Others	58	151	
**Do You Take Additional Supplements?**			
**Entity**	**Supplement Use**	**No Supplement Use**	***p*-Value**
Breast	12	5	**0.0021**
Others	61	138	
Hematology malignant	20	63	0.7527
Others	53	100	
Head–neck	5	38	**0.0012**
Others	67	105	
Lung	12	27	0.7121
Others	61	116	
Urooncology	3	14	0.1856
Others	70	129	

Significant *p*-values were depicted in bold.

**Table 4:** Diet changes und supplement use in cancer patients.

Cohort	Total [N]	Any Diet Change [N]	Preference [N]	Avoidance [N]	Supplements [N]
All	235	76	47	62	65
Radiation	97	14	6	11	25
Hema/Onco	102	45	32	35	36
Head–Neck	36	17	9	16	4

## Data Availability

The data presented in this study are available on request from the corresponding author.

## Appendix A

Screening tool combining NRS-2002 and the authors’ four-item questionnaire (German).

**NRS2002 (Nach Nach Kondrup J et al., Clinical Nutrition 2003; 22: 415–421** [12] **)**					
**Vorscreening**					
**Ist der Body Mass Index < 20,5 kg/m^2^?**					
Hat der Patient die letzten 3 Monate an Gewicht verloren					x
War die Nahrungszufuhr in der letzten Woche vermindert?					
Ist der Patient schwer erkrankt (z. B. Intensivtherapie)?					
**Screening**					
Störung des Ernährungszustandes	Punkte	+	Krankheitsschwere		Punkte
Keine	0		Keine		0
Mild Gewichtsverlust > 5% in 3 Monaten oder Nahrungszufuhr < 50–75% des Bedarfs in der Vorwoche	1		Mild Schenkelhalsfraktur, chronische Erkrankung mit Komplikationen, Krebsleiden		1
Mäßig Gewichtsverlust > 5% in 2 Monaten oder BMI 18,5–20,5 kg/m^2^ und reduzierter Allgemeinzustand oder Nahrungszufuhr 20–25% des Bedarfs in der Vorwoche	2		Mild Große Bauchchirurgie, Schlaganfall, Pneumonie, hämatologische Krebserkrankung		2
Schwer Gewichtsverlust >5% in 1 Monat oder BMI < 18,5 kg/m^2^ und reduzierter Allgemeinzustand oder Nahrungszufuhr 0–25% des Bedarfs in der Vorwoche	3		Schwer Kopfverletzung, Knochenmark-transplantation, Intensivpflichtige Patienten		3
+1 Punkt, wenn Alter ≥ 70 Jahre					
≥**3 Punkte**	**Risiko für Malnutrition liegt vor, Erstellung eines Ernährungsplans**				
<**3 Punkte**	**wöchentlich Screening wiederholen**				
**Haben Sie Ihre Ernährungsgewohnheiten verändert, seitdem Sie von Ihrer Krebsdiagnose wissen?**					
**Ja**					**Nein**
**Verzichten oder vermeiden Sie bestimme Nahrungsmittel?**	JA			NEIN	Bevor Sie mit einer Krebsdiöt beginnen, sprechen Sie bitte mit Ihrer/m HausärztIn oder behandelnde/n OnkologIn		
**Bevorzugen Sie bestimmte Nahrungsmittel?**	JA			NEIN			
**Nehmen Sie Nahrungsergänzungsmittel ein?**	JA			NEIN			
**Folgen Sie besonderen Ernährungshinweise/Diätplänen?**	JA			NEIN			
